# Patellar chondropathy: a brief overview of its history and prevalence

**DOI:** 10.1590/0100-3984.2021.54.1e1

**Published:** 2021

**Authors:** Marcelo Novelino Simão

**Affiliations:** 1 Radiologist at the Central Diagnóstico de Ribeirão Preto (CEDIRP), Physician in the Musculoskeletal Radiology Sector of the Hospital das Clínicas da Faculdade de Medicina de Ribeirão Preto da Universidade de São Paulo (HCFMRP- USP), Ribeirão Preto, SP, Brazil. Email: marcelo_simao@hotmail.com.

One of the pioneers in the study of the patellar cartilage, Dr. Ralph Edward Outerbridge, believed that condition known as chondromalacia patellae was more common than had previously been imagined. Outerbridge observed that, in the early stages of chondromalacia, the appearance of the patellar cartilage changes from healthy, bluish-white, shiny, and elastic to yellowish-white, soft, and swollen^(1)^.

Chondromalacia is a term of Greek origin, formed from a combination of the words *khóndros* (cartilage) and *malakos* (softened). It is widely used in the medical field in general and in a large number of scientific articles as a synonym for chondral lesion. However, it can be considered more of a figure of speech of the metonymy type, in which a thing or concept is referred to by the name of something closely associated with that thing or concept. In this case, it represents a whole spectrum of chondral changes, given that chondral lesions encompass much more than softening. As demonstrated by Outerbridge^(1)^, softening is only the initial stage of the chondral injury process. Therefore, terms such as chondropathy and chondral lesion better represent the spectrum of chondral involvement, as mentioned in the article by Krieger et al.^(2)^, published in the previous issue of **Radiologia Brasileira**.

There are several classifications for the assessment of chondral lesions, generally based on size, depth, tissue quality, and whether or not the subchondral bone is involved. The most traditional and well-known include that devised by Outerbridge^(1)^, whereas one of the most recent and detailed is the International Cartilage Repair Society (ICRS) classification^(2)^.

The gold standard for evaluating the articular cartilage of the knee is arthroscopy. Magnetic resonance imaging (MRI) has played a role in the assessment of cartilage, including that of the patella, since the early 1990s^(3)^. As a noninvasive method for diagnosing chondral lesions, MRI has produced varied and controversial results, those results being heavily dependent on the type of sequence used, field strength, degree of chondral injury and level of experience of the examiner. Another important point is that MRI can be used not only in the diagnosis and classification of chondral lesions but also in the evaluation of the repair process and postoperative evolution of such lesions^(4)^. It has been known for more than a decade that 3.0 T scanners perform better than do 1.5 T scanners, which in turn perform better than do those with even lower magnetic fields strength^(5)^. A systematic review and meta-analysis published in 2018 indicated that there is no significant difference between 1.5 T and 3.0 T scanners in terms of their performance in the evaluation of menisci and ligaments, although 3.0 T scanners were shown to be more accurate for the evaluation of cartilage^(6)^.

Finally, the diagnosis of patellar chondropathy and its classification based only on clinical symptoms of anterior knee pain are also controversial and inaccurate, which limits the indications for an invasive procedure such as arthroscopy^(7)^. In this context, of inaccurate clinical diagnoses, evaluation by noninvasive diagnostic methods that are more accurate, such as MRI, is gaining ground.

The study conducted by Krieger et al.^(2)^ took an approach different than that taken in most previous articles, because it emphasizes the identification of chondral lesions and more detailed mapping of their prevalence by gender, age, and body mass in high-field-strength (3.0 T) MRI scanners, thereby providing a better overview of the presentation of chondral lesions. In at least two respects, the results of their study are surprising. First, the authors found a high prevalence of chondral lesions in the patients under 30 years of age, although that prevalence was even higher in older age groups. Second, they found that the prevalence of advanced (ICRS grade 4) chondral injuries was quite high in the patients ≥ 40 years of age.

The Krieger et al.^(2)^ article will certainly be of interest to all radiologists whose work involves the interpretation of knee examinations, because this notion of the prevalence of patellar chondropathy and its classification is quite relevant. In addition, from a more scientific point of view, their findings may lay the groundwork for future studies aimed at clarifying the correlation between chondral lesions and clinical symptoms, as well as determining their population incidence and evaluating the patterns of their progression over time.
